# Do Calves Drink Water?

**DOI:** 10.3390/ani16070997

**Published:** 2026-03-24

**Authors:** Christophe Staub, Eric Venturi

**Affiliations:** INRAE Centre Val-de Loire, UE1297 Physiologie Animale de l’Orfrasière, 37380 Nouzilly, France

**Keywords:** water consumption, precision farming, animal welfare

## Abstract

Background: Today, it is important to measure livestock water consumption to devise sustainable solutions that consider environmental issues, livestock health requirements and animal welfare. Methods: This longitudinal study measured the water consumption of 66 calves subjected to two feeding diets: a recommended diet as control (CON) and an optimised diet (OPT). Individual measurements were collected daily and summarised on a weekly basis over a 20-week period. The analysis considered the impact of environmental conditions depending on the season of the calf’s birth. Results: Before weaning, calves spontaneously drank significant amounts of water in addition to the water brought by the calf milk replacer (CMR), but there was variability between animals. Water consumption among calves in the OPT group was higher than that among calves in the CON group from week 4 onwards (*p* = 0.005). At weaning, there was a significant increase in water consumption with a total water intake higher in calves in the OPT group compared to calves in the CON group (118.4 L and 78.9 L; *p* < 0.001). After weaning, water consumption was correlated with the solid feed intake in our model, which did not include direct fodder other than straw. There were no seasonal effects on water consumption before weaning at 9 weeks, but effects were observed after 13 weeks on the feeding plan (*p* = 0.008), with higher water consumption among calves born in winter and exposed to warmer temperatures in spring. Over a 20-week period, when calves had reached a weight of 180 kg in the OPT group and 150 kg in the CON group, water consumption had reached 1602 L and 1400 L respectively (*p* < 0.001). Conclusions: Free access to water should be maintained in calf rearing facilities, as water contributes to concentrated CMR and dry solid feed assimilation and the welfare of calves when the feeding plan remains at a modest level, enabling them to tolerate fluctuating environmental conditions.

## Simple Summary

Water consumption was determined in calves raised in a modern nursery equipped with connected drinking troughs that allow individual measurements. The calves were fed two diets consisting of milk-based feed until 9 weeks of age, followed by cereal-based feed after weaning, and unlimited straw. Water consumption differed between the two groups. Animals on the most generous feeding diet were also those that drank the most water spontaneously, despite having access to larger volumes of milk feed. However, water consumption varied between individuals, with some drinking virtually no water until weaning. The amount of water varied based on diet as well as environmental conditions, although there were no seasonal effects before weaning. Water consumption increased after weaning and during hot periods, but environmental parameters seemed to have a lesser impact on water consumption than feeding diet, even during the hot weeks at the end of spring and beginning of autumn. As the nursery was not equipped with active ventilation, no animals were in the building from mid-May to mid-September.

## 1. Introduction

In the context of sharing resources with other human activities, water consumption by agriculture has been a matter of debate [1]. The subject has become a major environmental issue, and more information is needed to understand the impact of livestock water consumption and to devise sustainable solutions that consider environment issues, livestock health requirements and animal welfare. In this context, it would be interesting to know whether calves drink water spontaneously and from what age in a modern rearing system equipped with automated individual water monitoring.

European legislation states that “animals must have access to fresh water from the age of two weeks, but water can be substituted for other liquids, except when it is hot or when animals are sick, conditions under which animals must have access to fresh water at all times” [2]. It remains unclear whether water can be replaced with milk or milk replacers if calves are healthy. It is also unknown at what age this practice become detrimental to the animals’ comfort or growth. In addition to this legislation, scientific literature on the subject is far from exhaustive. Scientists agree that water consumption is necessary to maintain homeostasis and eliminate waste [3,4], but when it comes to water intake in addition to milk or calf milk replacer (CMR) during the pre-weaning period, there are different schools of thought. Some say that water is necessary in addition to milk from birth [5,6], others only from three weeks [7] or only during the milk decline phase of the milk feeding plan [8], and others only after weaning [9]. Some say that water has no impact on dry matter intake (DMI) before 8 weeks [10] or even 10 weeks when using CMR [11], but it depends on the CMR concentration [8,11]. Still others say that growth is directly correlated with DMI even before weaning because it improves rumen growth and feed efficiency [5,6]. The hypothesis that ad libitum water can increase the risk of diarrhoea [12] has now long been disproved [13,14], and it is excessive feed intake that causes diarrhoea [15]. Water supply by administering aqueous solutions of oral electrolytes represents the cure [16,17], but it is not advisable to provide electrolytes directly in the CMR, as no additional water will be provided and the animals could risk fatal ionic imbalances [18]. An abundant supply of fresh and easily accessible drinking water is essential for maintaining animal health parameters [19]. It is relatively well established that water should be provided to calves as early as possible [20], even though water consumption is negligible before 2 weeks of age. Several studies have shown that there are no positive effects of water intake on animal growth before weaning [20,21,22,23] except during hot summer months [24]. There is little information available on spontaneous livestock water consumption delivered by water delivery systems with individual water intake monitoring. A comprehensive study was recently published on the subject [25], but it does not contain any data on calf consumption before weaning.

Regarding heat, authors agree that water intake depends on environmental conditions. Heat increases body fluid loss due to sweating and panting (increased respiratory rate). In hot weather, the effects of water restriction can quickly prove catastrophic, putting the animal’s life at risk even if cattle have a significant ability to adapt to water shortages by using the rumen as a water reservoir and regulating renal and intestinal water losses through peripheral osmoreceptors in the gastrointestinal and vascular systems [26]. The present study aimed to illustrate that calves spontaneously drink water from an early age and that their water consumption increases with solid feed intake. This study also aimed to measure the impact of seasonal variations in temperature and humidity on calf water consumption in relation to feeding practices.

## 2. Materials and Methods

The experimental set up was described in a previous study exposing the main zootechnical results [27]. Briefly, female Holstein calves born from sexed artificial insemination (AI) were fed whole milk from their mother for 2 meals per day for 72 h before switching to a CMR (Univor Premium™, Bonilait Protéines, Chasseneuil du Poitou, France; Supplementary Table S1) at the beginning of the fourth day. The solid ration consisted solely of dry solid feed (Floribelle™, Tellus, Saint Germain de Salles, France; Supplementary Table S1), introduced at 2 weeks. The animals had ad libitum access to straw and water throughout the experiment. In total, 66 calves were followed from birth to 20 weeks, corresponding to their departure from the calf nursery. Calves were organised in 4 blocks (2 blocks in summer (SUM) and 2 blocks in winter (WIN)), and animals were divided randomly into 2 groups (control (CON) and optimal (OPT)) in each block by taking care of their birth weight (Supplementary Table S2), their sire, the dam’s parity and the dam’s genetic score that was derived from the dam’s genotyping as already described [27]. Diets were qualitatively identical in both groups, with only the quantities varying. The CON and OPT treatment diets allowed calves to reach 200 kg in 7.2 and 5.4 months, representing an average growth from birth to 200 kg of 750 and 990 g/day, respectively. Individual spontaneous water consumption was measured daily with water supplying devices (La Buvette, Tournes, France). In the nursery, the flow rate of the drinking troughs was set to 1 L/min for calves from birth to weaning at 9 weeks, then at 2 L/min for calves aged 10–20 weeks. To streamline the analysis, the amount of water drunk was summarised on a weekly basis.

Since calves were not all born at the same time, each water consumption value was associated with the temperature and humidity at the time of drinking. Environmental measurements were expressed in terms of maximum temperature (MAX TEMP) and temperature and humidity index (THI) [28] to avoid making arbitrary choices regarding which values to use when calculating any means (temperatures can vary greatly between night-time, morning and afternoon) and to simulate the worst conditions the animals might have experienced.

Statistical analyses were carried out using SAS software (SAS version 9.4, SAS Institute Inc, Cary, NC, USA), and data are presented as the means ± standard error of the mean (SEM). The Gaussian distribution of each measure was assessed using Fisher tests. If the variances were equal, then bi-parametric analysis of variance (ANOVA) was performed to compare treatment groups, season blocks and interactions between treatment and season at a given age. Otherwise, non-parametric Kruskal–Wallis tests and Wilcoxon’s tests were performed. Given the number of tests, we applied a multiple testing *p*-value correction using the Benjamini–Hochberg procedure to control the false discovery rate across the weekly comparisons. When it was useful to track parameters over time, mixed procedures for repeated effects were used, including the animal as a random effect. For environmental data, differences in proportions between years were determined using chi-square tests. For all tests, the level of statistical significance was set at *p* < 0.05.

## 3. Results

Table 1 compares the spontaneous individual water consumption of calves fed with two treatment diets (CON and OPT), whose individual consumption of milk replacer volume and solid feed mass were specified. Water consumption was higher in the OPT group than in the CON group after the fourth week of the milk feeding plan onwards (*p* = 0.005) and throughout the 20 weeks of the study (*p* < 0.001). At weaning, calves in the CON and OPT groups spontaneously drank an average of 78.9 and 118.4 L of water, respectively, in addition to milk replacer, for which consumption was also higher in animals from the OPT group on all dates of the milk feeding plan (*p* < 0.001). After weaning, water consumption remained higher in calves in the OPT group, which also consumed greater quantities of concentrate feed, compared to those in the CON group (*p* < 0.001). Over the 20 weeks, the amount of water consumed spontaneously by the animals reached 1200 and 1602 L in CON and OPT groups, respectively, which represents a significant difference between the groups (*p* < 0.001). There was also a seasonal effect, with higher water consumption among calves born in WIN and exposed to warmer temperatures in spring at 13 weeks after birth compared to calves born in SUM and growing mainly during the cold seasons (*p* = 0.008). There were no significant seasonal effects on water consumption before weaning (*p* = 0.468).

**Figure 1:**
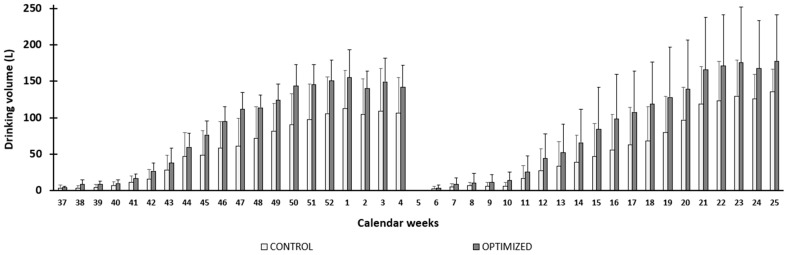
Evolution of water consumption in both control and optimised groups over time. Control treatment diet group (CON) with effective growth of 750 g/day from birth to 200 kg: white bars. Optimised treatment diet group (OPT) with effective growth of 990 g/day from birth to 200 kg: grey bars.

**Figure 2:**
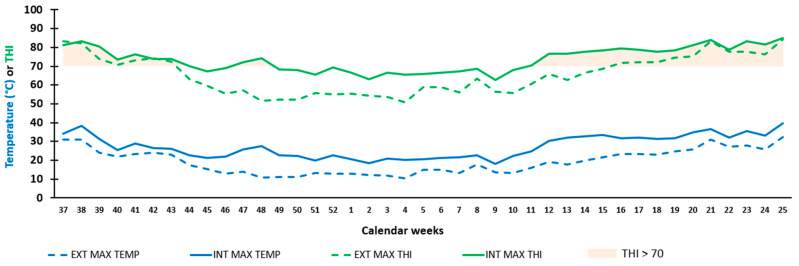
Evolution of environmental parameters inside and outside the calf rearing building over time. External maximum temperature (EXT MAX TEMP): blue dotted line. Internal maximum temperature (INT MAX TEMP): blue solid line. External maximum temperature and humidity index (EXT MAX THI): green dotted line. Internal maximum temperature and humidity index (INT MAX THI): green solid line. Internal maximum temperature and humidity index (THI) > 70: light red area.

Figure 1 illustrates the water consumption of the two groups of animals, CON and OPT, whereas Figure 2 shows the maximum outside and inside temperature (EXT MAX TEMP and INT MAX TEMP, respectively) and the reinforcing effect of humidity on temperature (EXT MAX THI and INT MAX THI). Both graphs summarise the monitoring of four blocks of calves over four years, with SUM blocks corresponding to animals born in September 2019 and 2021 and WIN blocks corresponding to animals born at the end of winter in February 2020 and 2022, which experienced climatically equivalent hot seasons (Table 2). There was an increase in water consumption over time (*p* < 0.001) and according to the season of birth (*p* < 0.001). Figure 2 shows the periods of the year when THI exceeded the threshold value of 70 in the nursery: at the end of summer and during autumn (weeks 37–48) and during spring (weeks 12–25).

Table 2 compares the number of weeks and average values for temperature and THI calculated from weeks during which the temperature reached at least 30 °C and during which the maximum THI was above 70 or 75 in 2019–2023. The only significant difference was the number of weeks during which the temperature reached at least 30 °C, which was only 5 weeks in 2021, significantly lower than in the other years of the study, except for 2020. The years 2020 and 2022, during which calves were born in winter and therefore raised during the warm spring period, were not significantly different.

## 4. Discussion

In our study, water consumption among calves in the OPT group was higher than that among calves in the CON group from week 4 onwards, at a time when the milk feeding plan was still in its ascending phase. At this point, in weeks 4 and 5, calves in the OPT group consumed 55.5–61.3 L of CMR per week, which corresponds to a drinking volume of 8–9 L per day. Despite these high liquid volumes, water consumption was significantly higher in OPT group calves, possibly due to the CMR concentration of 155 g/L, which was higher than that of CON group calves at 115 g/L, as shown by Davis and Drackley [8]. The difference between the two groups of animals was even more pronounced towards the end of the milk feeding period and the transition to solid feed, with solid feed consumption becoming significantly different between the two groups from week 8 onwards. The animals’ water consumption increased in week 9, when they only received one milk feed per day (instead of four) to encourage them to eat solid feed. At weaning, in addition to the 385 L of CMR at 155 g/L (corresponding to 59.7 kg of CMR), the calves in the OPT group drank nearly 120 L of water, which may have helped them assimilate the concentrated CMR to achieve an average growth of 860 g/day between birth and weaning [27]. Even with a lower performance growth of 650 g/day between birth and weaning, calves in the CON group drank 79 L of water in addition to the 343 L of CMR at 115 g/L. These results confirm that the water present in CMR is insufficient even when it is in low concentrations [20].

There are significant differences between individuals as shown by the gap between the minimum and the maximum in water consumption determined weekly, reflecting differences in behaviour when these are observed in calves within the same feeding group [29]. Calves have different personalities, some are playful, while others are more cautious. An animal rewarded with a positive experience gains confidence. When several calves are in the same straw-bedded area, as in our case, there is clearly a phenomenon of imitative behaviour, with the timid animals learning from the experiences of the more daring ones. Broadening the topic, the rearing method can also influence the animals’ behaviour regarding water consumption, as it has been previously stated [30]. Some calves drank almost nothing before week 9 (1.4 L during week 8), which corresponds to weaning in our system. At the same time, the calves that consumed the most water drank 56 L per week, i.e., 8 L per day. Even at weaning, there was still a ratio of 1–4 between the extremes. This difference between the extremes persisted after weaning in our system, as the animals only had access to concentrate and straw, which are known for their low water content compared to fresh grass or silage [24].

There were no seasonal effects in our modern nursery building before weaning, meaning that calves born at the end of summer did not feel the need to drink water due to the heat. This could be different in other, more spartan housing conditions, such as calf hutches, for example. The lack of measurements during peak summer months is one limitation of our study, as is the absence of physiological hydration markers.

The post-weaning part of the study showed a logical increase in water consumption proportional to the solid food intake. At 20 weeks, at the age when the animals had reached a weight of 150 kg in the CON group to 180 kg in the OPT group, water consumption had reached 1200 and 1602 L, respectively. Within each group, there was no correlation between water intake and growth. The effect of diet on water consumption represented a 33% increase (from 1200 to 1602 L), which was greater than the effect of season on water consumption, representing an increase of only 18% (from 1268 to 1492 L) as previously reported [31]. These results were obtained with no animals in the nursery from mid-May to mid-September, i.e., during the hottest months. If this were not the case, the building would need to be equipped with active ventilation, as we have done in the heifer and dairy cow buildings.

## 5. Conclusions

This study confirms that calves spontaneously drink water before weaning, which might contribute to a need for better CMR assimilation, especially when it is concentrated to achieve high growth. Even though the farming practices of this study enhanced calves’ attraction for water (high lactating feeding plan and CMR concentration, the use of straw as the sole source of direct fodder after weaning) and there were variations in behaviour among individuals, calves drank water spontaneously, and this consumption was not harmful to their health. We conclude that, in addition to being a comfort factor, water consumption by calves does not jeopardise their growth performance. In contrast, water is an important component of the nutrition plan when associated with concentrated CMR and high levels of solid feed with the aim of obtaining average growth rates of 1000 g/day, which was the condition of calves from the OPT group in this study. The amount of water drunk directly depended on the solid feed intake after weaning. The effect of temperature further increased the amount of water drunk, regardless of diet. Despite the construction of a modern nursery building that is particularly well insulated and is well above standard comfort levels for livestock, uncomfortable conditions can occur from spring to autumn, and in exceptional cases, THI can exceed 70 during the late autumn months when a number of adverse factors converge (such as mild temperatures, soiled litter, maximum humidity on rainy days and limited passive ventilation in the total absence of wind). In conclusion, unrestricted access to water is mandatory, even under high milk allowances.

## Figures and Tables

**Table 1:** Quantity of milk replacer, solid feed and water consumed by calves in this study.

Weeks	CMR ^1^ (L/wk)			Solid Feed ^2^ (kg/wk)			Water ^3^ (L/wk)								Probability ^4^		
	CON ^5^	OPT ^6^	SEM ^7^	CON	OPT	SEM	CON	OPT	SEM	WIN ^8^	SUM ^9^	SEM	MIN	MAX	TGE	SE	TGE × SE
1	16.5	**18.7**	2.6				1.0	0.5	1.3	0.6	0.7	1.0	0.0	3.0	0.920	0.681	0.748
2	34.8	**41.3**	1.9				1.4	1.7	1.7	1.7	1.5	2.0	0.0	5.0	0.420	0.991	0.880
3	41.7	**47.8**	3.2				2.3	4.6	5.2	2.3	4.2	4.8	0.0	24.1	0.120	0.144	0.430
4	48.7	**55.5**	2.6	0.7	0.8	0.5	3.1	**6.1**	4.3	3.8	5.1	4.5	0.0	18.2	**0.005**	0.185	0.693
5	55.7	**61.3**	4.5	1.0	0.9	0.6	5.8	**10.1**	7.2	7.7	7.7	8.0	0.1	29.9	**0.013**	0.991	0.880
6	54.8	**61.3**	1.9	1.3	1.3	0.5	7.6	**12.8**	9.0	9.8	9.9	8.3	0.1	42.3	**0.004**	0.991	0.880
7	41.7	**48.4**	0.6	2.7	2.7	0.7	9.9	**17.2**	9.9	11.8	14.3	9.0	0.5	44.8	**0.002**	0.326	0.991
8	33.9	**36.8**	1.3	5.7	**6.2**	0.4	14.5	**22.8**	10.6	15.9	20.2	10.6	1.4	56.6	**0.002**	0.130	0.880
9	13.9	**13.5**	1.3	8.6	**11.3**	0.8	33.3	**42.6**	13.5	35.0	39.7	12.7	17.3	70.1	**0.003**	0.202	0.880
Total weaning	342.6	**385.2**	12.3	20.0	**23.2**	4.2	78.9	**118.4**	4.3	88.6	103.3	4.0	78.9	118.4	**0.001**	0.468	0.936
10				11.7	**18.4**	1.9	53.7	**65.9**	20.9	58.2	59.8	22.9	21.0	144.7	**0.029**	0.991	0.880
11				12.7	**23.1**	0.6	61.6	**84.6**	22.4	71.4	71.9	27.5	27.5	144.6	**0.003**	0.991	0.991
12				14.3	**25.2**	1.6	75.8	**104.8**	26.9	95.2	82.3	28.4	42.0	176.1	**0.002**	0.071	0.880
13				15.7	**26.3**	2.2	86.2	**124.5**	29.9	**113.4**	93.2	32.6	36.3	184.9	**0.002**	**0.008**	0.991
14				16.6	**27.4**	1.9	96.2	**131.6**	31.7	**122.0**	102.1	33.8	41.3	209.1	**0.002**	**0.014**	0.991
15				16.8	**28.0**	1.6	105.9	**140.3**	36.7	**132.0**	110.7	35.6	52.7	245.6	**0.002**	**0.015**	0.991
16				17.1	**29.0**	0.8	111.3	**154.4**	38.9	**143.4**	117.8	38.6	53.6	244.1	**0.002**	**0.008**	0.880
17				17.2	**29.1**	0.9	123.0	**164.7**	38.6	**153.7**	129.7	40.2	76.1	239.7	**0.002**	**0.013**	0.880
18				17.5	**30.3**	0.9	130.0	**169.9**	35.9	**163.0**	133.0	36.2	83.8	238.0	**0.002**	**0.007**	0.748
19				18.0	**31.3**	0.8	136.8	**169.8**	44.7	**172.0**	131.8	40.6	71.3	257.0	**0.002**	**0.007**	0.220
20				18.2	**32.7**	1.0	141.1	**172.8**	45.1	**178.8**	132.7	41.3	79.4	260.5	**0.002**	**0.007**	0.880
Total 20 weeks				272.7	**468.1**	12.9	1200	**1602**	14.9	**1492**	1268	14.7	1200	1602	**0.001**	**0.001**	0.700

^1^ Univor Premium™ (Bonilait Protéines, Chasseneuil du Poitou, France) used as calf milk replacer (CMR). ^2^ Floribelle™ (Tellus, Saint Germain de Salles, France) used as dry solid feed. ^3^ Water was not restricted, but individual consumption was measured with La buvette electronic drinking troughs (Tournes, France). ^4^ Probability of treatment group effect (TGE) or season effect (SE) or their interaction. We applied a multiple testing correction using the Benjamini–Hochberg procedure to control the false discovery rate across the weekly comparisons. ^5^ Control group (CON): total milk feeding plan carried out over 9 weeks with 39.4 kg of CMR prepared at a concentration of 115 g/L. ^6^ Optimised group (OPT): total milk feeding plan carried out over 9 weeks with 59.7 kg of CMR prepared at a concentration of 155 g/L. ^7^ SEM: greatest value of the standard error of the mean within the group. ^8^ Calves born in winter (WIN), i.e., main growth during a hot season. ^9^ Calves born in summer (SUM), i.e., main growth during a cold season. Numbers in bold represent averages that are significantly different with a *p*-value < 0.05.

**Table 2:** Climate conditions from 2019 to 2023.

Years	Temperature Max > 30 °C					THI Max > 70			THI Max > 75		
	N Weeks ^1^	Min ^2^	Mean ^3^	Max ^4^	SEM ^5^	N Weeks ^6^	Mean ^7^	SEM ^5^	N Weeks ^6^	Mean ^7^	SEM ^5^
2019	14 ^a^	9.9	20.5	33.3	3.6	24 ^a^	80.8	7.3	17 ^a^	84.2	5.7
2020 *	10 ^ab^	10.7	20.9	33.9	3.0	27 ^a^	79.5	6.4	18 ^a^	83.0	4.8
2021	5 ^b^	12.6	20.7	32.0	1.7	25 ^a^	78.0	5.6	16 ^a^	81.3	4.1
2022 *	13 ^a^	11.9	21.8	35.0	3.1	26 ^a^	81.4	7.5	19 ^a^	85.0	5.1
2023	14 ^a^	11.3	20.6	32.1	2.7	23 ^a^	82.0	5.0	21 ^a^	82.9	4.2

^1^ Number of weeks when the maximum temperature exceeded 30 °C. ^2^ Average minimum temperature during weeks when the maximum temperature exceeded 30 °C. ^3^ Average mean temperatures during weeks when the maximum temperature exceeded 30 °C. ^4^ Average maximum temperatures during weeks when the maximum temperature exceeded 30 °C. ^5^ SEM: greatest value of the standard error of the mean within weeks. ^6^ Number of weeks when the maximum temperature and humidity index (THI) exceeded 70 or 75. ^7^ Average maximum THI during weeks when the maximum THI exceeded 70 or 75. * Years during which calves born in winter were reared during the warm periods in spring (see Figure 1). ^ab^ Values with different lowercase letters are different at *p* < 0.05.

## Data Availability

Data are held in the Research Data French Government depository allowing sharing of research data to the international community. When the manuscript has been accepted, data will be available without any restriction at the following public URL: https://doi.org/10.57745/FJFEJN.

## Supplementary Materials

The following supporting information can be downloaded at: https://www.mdpi.com/article/10.3390/ani16070997/s1. Supplementary Table S1: Nutritional values of the feeds used in this study. Supplementary Table S2: Calves born during this study.

## Abbreviations

The following abbreviations are used in this manuscript:

CON	Control group
OPT	Optimised group
SUM	Summer blocks
WIN	Winter blocks
CMR	Calf milk replacer
DMI	Dry matter intake
WI	Water intake
MIN	Minimum
MAX	Maximum
TEMP	Temperature
THI	Temperature and humidity index
