# Supplementary figures and images for: Do Calves Drink Water?
Source: Animals (Basel). 2026 Mar 24;16(7):997. doi: 10.3390/ani16070997 (PMC13071965; doi:10.3390/ani16070997)

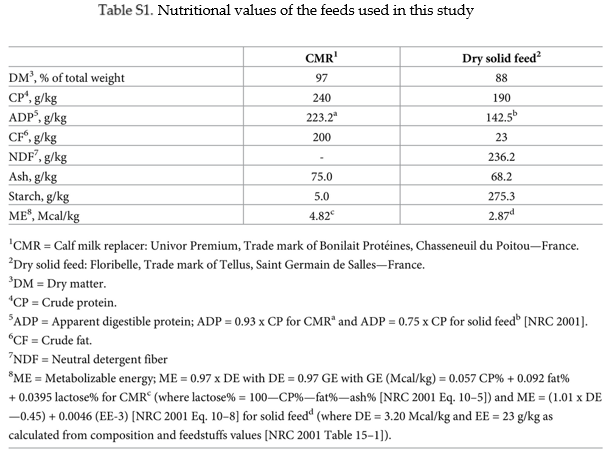

Supplement: Supplementary file 1 [file animals-16-00997-s001.zip › Supplementary_table_S1.png]

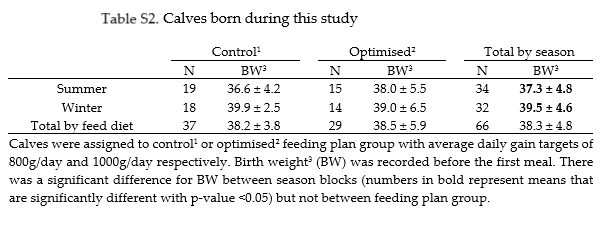

Supplement: Supplementary file 1 [file animals-16-00997-s001.zip › Supplementary_table_S2.png]
